# Risk surveillance and mitigation: autoantibodies as triggers and inhibitors of severe reactions to SARS-CoV-2 infection

**DOI:** 10.1186/s10020-021-00422-z

**Published:** 2021-12-20

**Authors:** Catherine Chen, Aisah Amelia, George W. Ashdown, Ivo Mueller, Anna K. Coussens, Emily M. Eriksson

**Affiliations:** 1 The Walter and Eliza Hall Institute of Medical Research, 1G Royal Parade, Parkville, VIC 3052 Australia; 2 Department of Medical Biology, The University of Melbourne, Melbourne, VIC 3052 Australia; 3 Department of Pathology, Institute of Infectious Disease and Molecular Medicine, Wellcome Centre for Infectious Diseases Research in Africa, University of Cape, Cape Town, South Africa

**Keywords:** Autoantibodies, SARS-CoV-2, COVID-19, Autoimmunity

## Abstract

COVID-19 clinical presentation differs considerably between individuals, ranging from asymptomatic, mild/moderate and severe disease which in some cases are fatal or result in long-term effects. Identifying immune mechanisms behind severe disease development informs screening strategies to predict who are at greater risk of developing life-threatening complications. However, to date clear prognostic indicators of individual risk of severe or long COVID remain elusive. Autoantibodies recognize a range of self-antigens and upon antigen recognition and binding, important processes involved in inflammation, pathogen defence and coagulation are modified. Recent studies report a significantly higher prevalence of autoantibodies that target immunomodulatory proteins including cytokines, chemokines, complement components, and cell surface proteins in COVID-19 patients experiencing severe disease compared to those who experience mild or asymptomatic infections. Here we discuss the diverse impacts of autoantibodies on immune processes and associations with severe COVID-19 disease.

## Background

COVID-19 clinical course varies considerably between individuals, ranging from asymptomatic, mild/moderate and severe disease, with fatal or long-term sequela in the worse cases. Symptoms associated with mild or moderate disease include fever, cough, shortness of breath, fatigue, muscle aches, headaches, gastrointestinal upset, loss of taste or smell, with emerging SARS-CoV-2 variants also associated with a runny nose and sore throat (Sanyaolu et al. 2020; Study ZC 2021). Those who progress to severe disease can develop hypoxia and dyspnea, related to pneumonia and pulmonary oedema, and the most critical cases are associated with development of coagulopathy, respiratory failure, septic shock, stroke and/or multi organ failure (Wu and McGoogan 2020). More recently long COVID, a condition associated with oscillating symptomatic episodes recurring for weeks to months after acute infection, has been identified as an official diagnosis and can occur in individuals irrespective of disease severity during the acute phase (Greenhalgh et al. 2020; Huang et al. 2021).

Identifying immune mechanisms behind severe disease development informs screening strategies for risk surveillance, to enable faster triage of patients at highest risk of deterioration at initial diagnosis and inform development of targeted therapeutics to mitigate severe disease progression. However, to date clear prognostic indicators of individual risk of severe or long COVID remain elusive. Numerous meta-analyses have identified consistent associations between individual demographics, pre-existing health conditions and severe COVID-19 prognosis. Males and the elderly were more inclined to develop severe COVID-19 disease with the original SARS-CoV-2 variant (Sanyaolu et al. 2020; DelSole et al. 2020; Fang et al. 2020), as were individuals with co-morbidities (including hypertension, type 2 diabetes, chronic kidney disease (CKD), chronic obstructive pulmonary disease (COPD), and coronary heart disease (Fang et al. 2020; Guan et al. 2020; Zhao et al. 2020) and co-infections (including tuberculosis, and HIV-1) (Boulle et al. 2020). Whilst it is unlikely that the same mechanism of increased risk exists for the diversity of risk factors identified, understanding pre-existing immune dysregulation associated with these conditions provides insight into the contributing triggers for rapid deterioration once infected with SARS-CoV-2.

Given that autoantibodies (auto-Abs) contribute to interference of normal immune system functionality, it emphasizes the importance in investigating autoantibody presence within severe COVID-19 patients. Emerging evidence from case reports and cohort studies have so far detected a diverse range of auto-Abs more frequently present in serum/plasma of individuals who have developed severe COVID-19 disease, including those targeting cytokines, complement components, and coagulation factors (Bastard et al. 2020; Wang et al. 2021). These have the capacity to alter normal immune function such as viral clearance, cellular recruitment and immunoreceptor signalling (Wang et al. 2021).

**Fig. 1:**
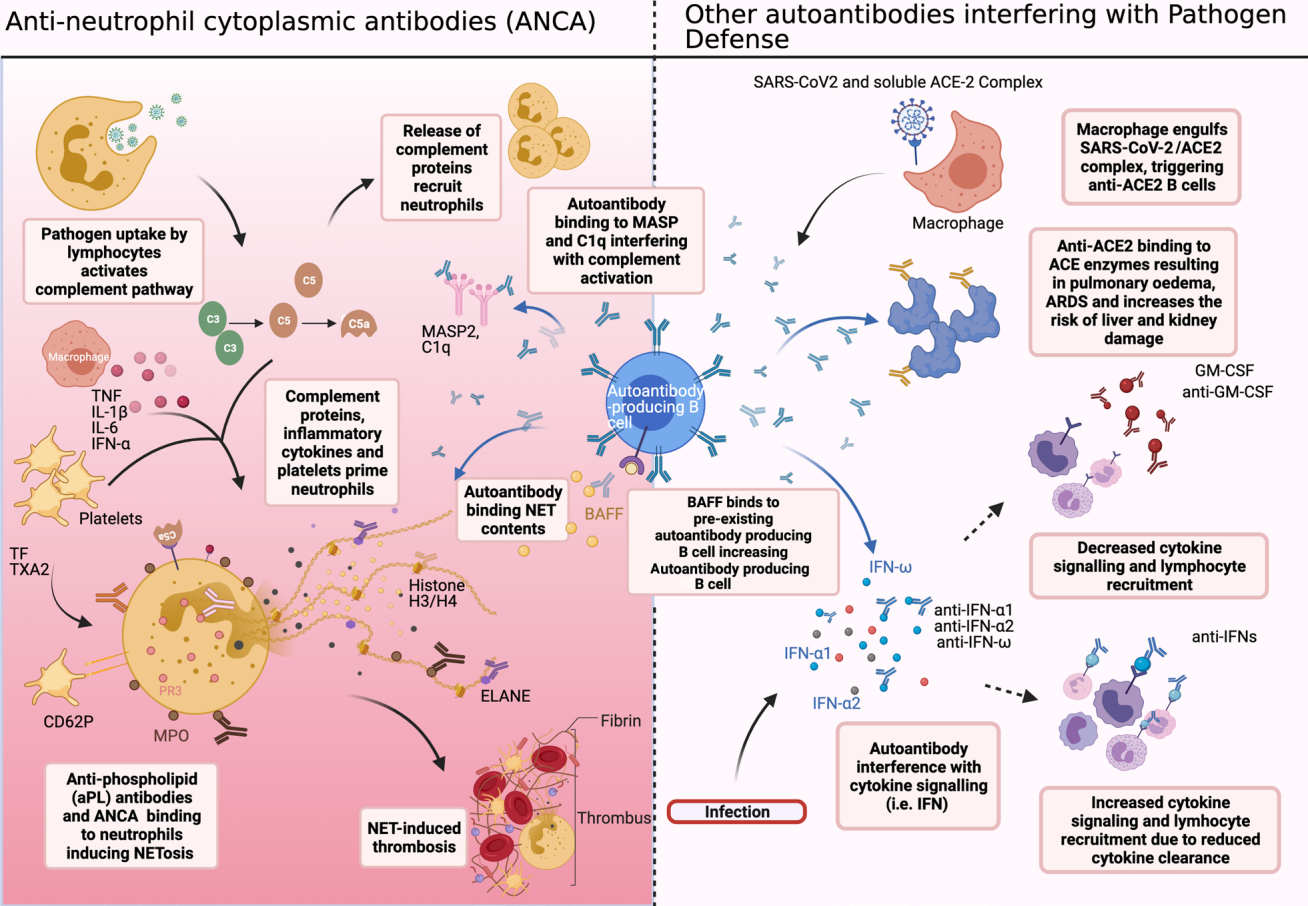
Potential processes affected by presence of autoantibodies in COVID-19. Left panel: pathogen uptake results in release of inflammatory markers and complement proteins which lead to neutrophil recruitment, activation and translocation of autoantigens. Anti-neutrophil cytoplasmic antibodies (ANCA) (anti-MPO, anti-PR3, anti-ELANE, and anti-aPL autoantibodies and anti-H3/H4), bind autoantigens and promote NETosis which induces a thrombotic response. NET contents can be recognised by anti-MPO, anti-H3/H4 and anti-ELANE autoantibodies. Autoantibodies to complement proteins (anti-MASP2, anti-C1q) interfere with complement activation. Right panel: autoantibodies bind to the B cell activating factor (BAFF) which enables production of more autoantibodies by pre-existing autoantibody producing B cells. Autoantibodies which interfere with pathogen defence include antibodies to complement, tissue antigens and cytokines which can disrupt cytokine communication (anti-GM-CSF) and cytokine clearance (anti-IFNs). SARS-CoV-2 bound to soluble ACE2 complex can be phagocytosed by macrophages and presented on the surface, inducing anti-ACE2 antibodies. Anti-ACE2 can bind soluble ACE2, reducing its capacity to act as a decoy for SARS-CoV-2, as well as have cross-reactivity with surface attached ACE, triggering further detrimental inflammation. Created with BioRender.com

**Fig. 2:**
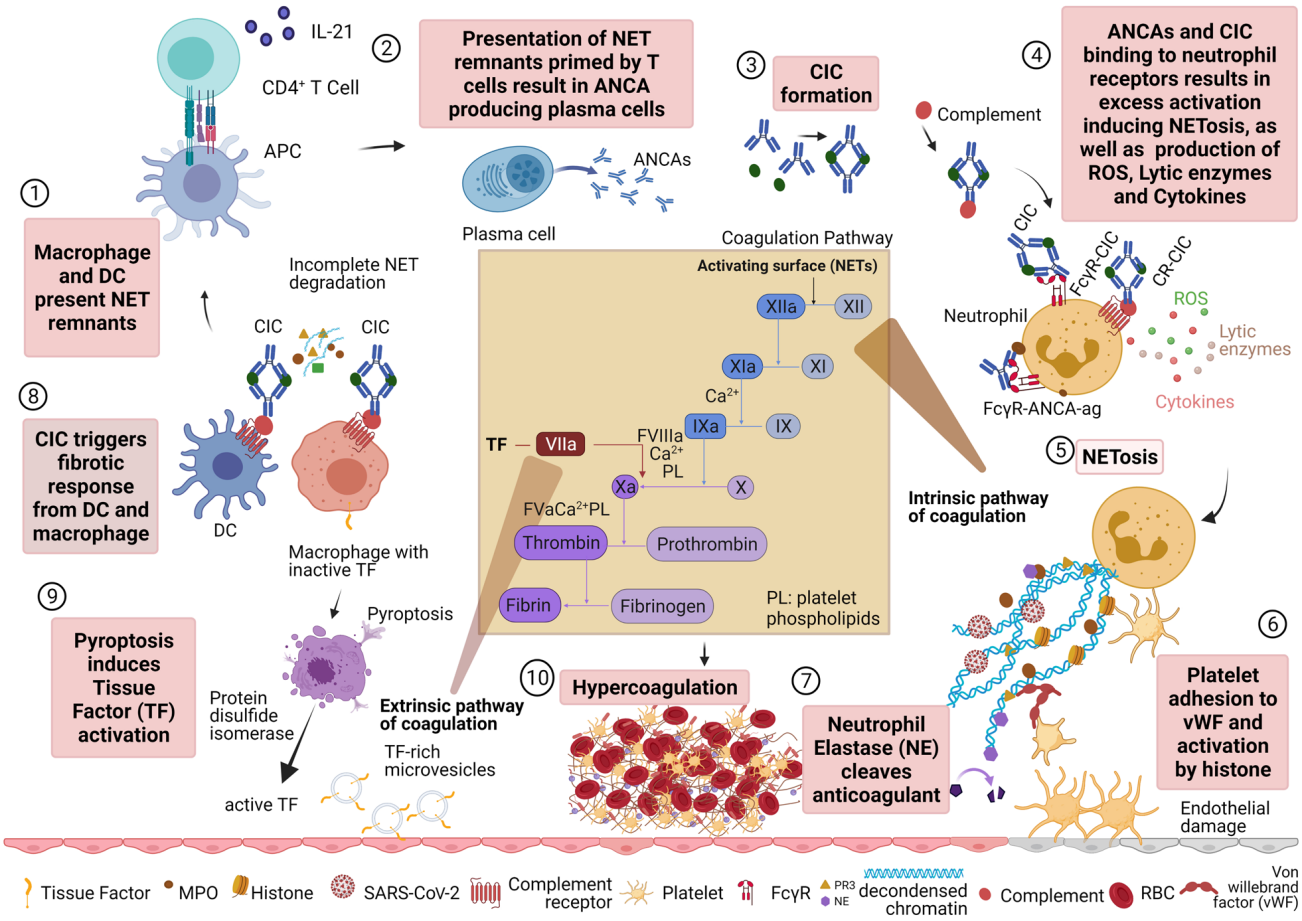
Incomplete NET degradation leads to hypercoagulation. (1) Incomplete NET degradation causes macrophage and dendritic cells (DCs) to present NET components to CD4 + T cells. (2) The T cells release IL-21 inducing differentiation of B cells into plasma cells to induce autoantibody production. (3) Anti-neutrophil cytoplasmic antibodies (ANCAs) can form circulating immune complex (CIC). (4) ANCAs and CIC activate neutrophils through FcɣR and complement receptor binding. Hyperactivation of neutrophils by autoantibodies produces Reactive Oxygen Species (ROS), cytokines storms, lytic enzymes, and NET. (5) NETosis releases decondensation of chromatin constructed from unwound neutrophil DNA and histones coated with neutrophil enzymes such as neutrophil elastase (NE), myeloperoxidase (MPO), and proteinase 3 (PR3). (6) Von Willebrand Factor (vWF) attracts platelets and histones activate them leading to the intrinsic pathway of coagulation. (7) NE cleaves anticoagulant which contributes to more coagulation. (8) CIC can trigger a fibrotic response from DCs and macrophages. (9) Macrophages carry inactive tissue factors which will be activated by pyroptosis, specifically by protein disulfide isomerase. Pyroptosis results in microvesicles that contain active tissue factors that lead to the extrinsic pathway of coagulation. (10) The vicious cycle of uncontrolled neutrophils activation and incomplete net degradation causes hypercoagulation through intrinsic and extrinsic pathways. Created with BioRender.com and adapted from Bautista-Becerril et al. (2021) and Jayarangaiah et al. 2020)

The function of auto-Abs could be protective or pathologic, depending on the immune pathways they perturbed. Their association with underlying co-morbidities identified to be associated with severe COVID-19 also suggests pre-existing serum levels and autoreactive T and B cells contribute to rapid deterioration following primary exposure to SARS-CoV-2. Patients who develop critical illness are found to have larger antibody-secreting cell expansion and extrafollicular B cell activation, commonly seen in autoimmune conditions (Woodruff et al. 2020). Prolonged elevation of auto-Abs following viral clearance could also contribute to the chronic symptoms associated with long COVID. Here we discuss the functional categories of auto-Abs associated with different COVID-19 disease phenotypes (Fig. 1) and the pathogenic and protective immune mechanisms they may potentiate (Fig. 2).

### Autoantibodies interfering with antiviral response capacity

The role of auto-Abs in the development of severe COVID-19 was first highlighted through two parallel papers. These describe inborn genetic errors in regions regulating type 1 IFN stimulated gene (ISG) expression controlled by TLR3 and IRF7 which were associated with severe risk and that patients who developed critical COVID-19 pneumonia were more likely to have high serum/plasma levels of IFN-α2 and IFN-ω neutralising auto-Abs (Bastard et al. 2020; Zhang et al. 2020a). In an additional study, a broad screen for auto-Abs that profiled 2770 auto-Abs to extracellular and secreted protein (the exoproteome), also found a smaller cluster of patients that displayed auto-Abs to cytokines, chemokines and type I (IFN-α2 and IFN-ω) and IIII IFN (IFNλ2 and IFNλ3), a signature highly correlated with disease severity (Wang et al. 2021). The study by Wang et al. (2021) also found that auto-Ab to GM-CSF, CXCL1 and CXCL7 antagonised their respective cytokine signalling pathways, which have key functions in regulating anti-viral immunity, whilst auto-Abs to CD38 and CD3ε increased antibody-mediated cellular phagocytosis causing immune-cell depletion.

Auto-Abs against cytokines and in particular to type I IFNs, have reproducibly been observed more frequently in patients with severe COVID-19 compared to mild, asymptomatic and healthy individuals (Bastard et al. 2020; Wang et al. 2021; Zhang et al. 2020a; Wijst et al. 2021). Bastard et al. (2020) found amongst a cohort of 987 patients who developed critical COVID-19, that 135 patients had detectable auto-Abs to IFN-α2 and/or IFN-ω. Of these patients, 49 patients had both IFN-α2 and IFN-ω auto-Abs, 45 individuals had anti-IFN-α2 and 41 had anti-IFN-ω. Moreover 15 of these patients also had detectable levels of auto-Abs to IFN-γ, GMCSF, IL-6, IL-10, IL-12p70, IL-22, IL-17A, IL-17F, and/or TNFβ (Bastard et al. 2020). However, of these additional auto-Abs they found only anti-IL-12p70, anti-IL-22, and anti-IL-6 were cytokine neutralizing. In a cohort of 194 individuals that experienced a range of disease, auto-Abs to IFN-ω and IFN-α2 were also found to have a longer duration of hospitalisation, suggesting they contribute to impaired viral clearance impacting disease course (Wang et al. 2021).

Auto-Abs against type I IFNs were observed to prevent activation of the TLR3 pathway, inhibiting the production of interferon stimulated genes such as CXCL10 which is important for antiviral responses (Bastard et al. 2020). The presence of auto-Abs against type I IFN, were confirmed to dampen tissue-specific antiviral IFN-I immunity in critically ill COVID-19 patients at the time of ICU admission (Lopez et al. 2021). Of the 26 critically ill patients tested, eight had IFN-ω and/or IFN-α2 auto-Abs detectable in serum and four of these also had auto-Abs detectable in nasal swabs. The five patients that had both IFN-ω and IFN-α2 in serum displayed low nasal IFN-I/III ISG scores in the nasopharyngeal mucosa (Lopez et al. 2021). Collectively, these findings suggest that defects in type 1 IFN responses decreases anti-viral immunity and increases severe COVID-19 development.

### Autoantibodies affecting complement and ACE2 functions

The complement cascade is another important innate immunity anti-viral mechanism, comprising three distinct, but converging, pathways (classical, lectin and alternative) which culminate in C3 proteolysis to the tertiary effectors C3a and C3b. Complement activation occurs early in the disease course before initiation of the cytokine storm, remaining elevated during serve stages in ICU and most elevated in those who die or experience thromboembolism (Nooijer et al. 2021; Holter et al. 2020). C1q is the first trigger of the classic pathways by binding to IgG and IgM to form immune complexes as well as binding CRP (McGrath et al. 2006). Anti-C1q has been found to be elevated in hospitalised COVID-19 patients and CRP is routinely elevated in patients who have severe and/or fatal outcomes (Wang et al. 2021).

The complement cascade can be directly activated by SARS-CoV-2 proteins where the lectin pathway recognition molecules MBL, FCN2 and CL-11 bind to the nucleocapsid (N) and Spike (S) proteins and their proteolytic complex partner MASP-2 also directly binds the N protein, promoting C4 cleavage (Ali et al. 2021). Additionally, S, but not N, protein activates the alternative complement pathway via binding to heparin sulphate on cell surfaces (Yu et al. 2020). Recombinant anti-MASP2 (Narsoplimab), which inhibits lectin pathway activation, has been tested as a COVID-19 therapeutic, resulting in rapid reduction in inflammatory and cell death markers (CRP, IL-6, IL-8, LHD) in six treated patients with ARDS (Rambaldi et al. 2020). As such, individuals with high levels of anti-MASP2 may be protected from serve COVID-19. However, maintained high levels of auto-Ab that interrupt complement-mediated cytotoxicity could decrease capacity to control other pathogens that are encountered and render individuals at risk in future pandemics. Emerging reports have also found auto-Ab generation towards ACE2, the primary receptor utilised by SARS-CoV-2 for cellular entry (Casciola-Rosen et al. 2020; Rodriguez-Perez et al. 2021). In humans, ACE2 can be found both attached to the endothelium as well as soluble in plasma, where the soluble forms serve as decoys to reduce SARS-CoV-2 entry (McMillan and Uhal 2020). It was first hypothesized that auto-Ab generation towards ACE2 may only hinder the function of ACE2 as decoys. However, the potential that immune complexes containing ACE2 may be phagocytosed by antigen-presenting cells (APCs) and presented for auto-Ab generation, which increases the risk of excessive inflammation, is now also recognised (McMillan and Uhal 2020).

Although both IgM and IgG anti-ACE2 have been observed in COVID-19 patients, it is IgM anti-ACE2 that are associated with severe disease and appear late in the disease course around day 8–10 of hospitalisation (Casciola-Rosen et al. 2020; Rodriguez-Perez et al. 2021). Anti-ACE2 can also have cross-reactivity with surface attached ACE. It is suggested that binding to surface ACE and ACE2 could trigger severe inflammation and endothelial damage, leading to ARDS and increase the risk of liver and kidney damage. (McMillan and Uhal 2020).

### Autoantibodies and neutrophil-mediated immune pathology

Neutrophils, as the first responders of the innate immune system were initially considered to be a homogeneous population due to their short life span (Pillay et al. 2010; Tak et al. 2013). In the last few decades, phenotypic and functional heterogeneity in neutrophils have been identified in healthy individuals and in relation to several diseases (Scapini et al. 2016) including COVID-19 (Lourda et al. 2021) where dysregulated neutrophil composition and activation is associated with severe COVID-19 disease.

Neutrophils are categorized and sorted into normal-density neutrophils (NDNs), the predominant type in healthy individuals (Ng et al. 2019), and low-density neutrophils (LDNs) frequently related to autoimmune and inflammatory diseases (Scapini et al. 2016; Silvestre-Roig et al. 2016). NDNs mainly consist of mature neutrophils, while both immature and mature neutrophils coexist in LDNs. LDNs and NDNs have thus far been identified to be either immunosuppressive or pro-inflammatory. Immunosuppressive neutrophils inhibit T cell proliferation through arginine-1 or reactive oxygen species (ROS) production and are commonly associated with pregnancy, systemic inflammation, and cancers (Silvestre-Roig et al. 2016). A high frequency of LDNs and irregular immature neutrophil have now also been associated with severe COVID-19 disease (Lourda et al. 2021; Schulte-Schrepping et al. 2020).

Mature neutrophils are derived from granulocyte-monocyte progenitors (GMP) which undergo several developmental stages in the bone marrow before being released into circulation (Ng 2019; Reusch et al. 2021). Changing expression of chemokine receptors from CXCR4 to CXCR2 determines the release of mature neutrophils from bone marrow (Reusch et al. 2021). In circulation, neutrophil heterogeneity is related to cell age, with older neutrophils exhibiting lower CD62L expression and increased complement receptor 3 (CD11b) and CXCR4 expression compared with freshly released neutrophils. Increased CXCR4 on aged neutrophils result in migration to the bone marrow, liver, or spleen where they are phagocytosed by macrophages. However, inflammation leads to emergency granulopoiesis with increased emergence of immature neutrophils from the bone marrow into circulation, further altering neutrophil heterogeneity in severe COVID-19 patients (Ng 2019; Reusch et al. 2021; Manz and Boettcher 2014).

Pro-inflammatory LDNs are commonly associated with autoimmune diseases, such as anti-neutrophil cytoplasmic antibody (ANCA)-associated vasculitis (AAV). ANCA are auto-Abs which target neutrophil antigens, such as myeloperoxidase (MPO) and proteinase 3 (PR3), and when bound to these autoantigens, triggers self-destruction of neutrophils via neutrophil extracellular traps (NET) formation (NETosis) (Flint et al. 2015; Nakazawa et al. 2019). NETosis is a process during which neutrophils release their DNA by decondensing chromatin as a means to trap and kill extracellular pathogens in a web-like structure. Dysregulated and persistent NETosis, as found in AAV patients (Nakazawa et al. 2019) is caused by low DNase activity and high NETosis. This is accompanied by increased ANCA concentrations in sera, where more ANCA are generated as dendritic cells phagocytose NETs and present components to activate autoreactive T and B cells (Nakazawa et al. 2019) (Fig. 2). Accumulation of NETs in lung tissue of severe COVID-19 patients co-localised with coagulation factors resulting in thromboinflammation, was found to be partially due to defective NET clearance by DNAses (Englert et al. 2021; Middleton et al. 2020).

### Autoantibodies and vascular coagulation

Arterial and venous blockage due to thrombosis-driven coagulation has also been a hallmark of severe COVID-19 (Middleton et al. 2020) and the rare severe thrombotic reactions to adenovirus-based COVID-19 vaccines (Greinacher et al. 2021; Scully et al. 2021). Auto-Abs which promote blood vessel blockages, include another ANCA subtype of anti-phospholipid antibodies (aPLs), which includes anti-cardiolipin, anti-prothrombin and anti–β2 glycoprotein IgG, IgM and IgA. aPLs bind to neutrophil and other cell surfaces promoting platelet aggregation and thrombosis and have been identified more frequently in severe COVID-19 patients (Zhang et al. 2020b; Zuo et al. 2020). These auto-Abs accelerate thrombosis via triggering of NETosis, with NETs trapping platelets, red blood cells and forming a scaffold for thrombus formation, ultimately resulting in coagulopathy and a pro-thrombotic risk (Fig. 2).

The propensity of coagulopathy associated with SARS-CoV-2 infection, has resulted in the WHO recommending treatment with the blood thinning anticoagulant heparin (Organization 2021). However, recent reports have found associations between COVID-19 patients and the development of heparin-induced thrombocytopenia (HIT), a condition associated with autoimmunity caused by platelet-activating anti-platelet factor 4 (PF4) antibodies forming an immune complex with heparin (Brodard et al. 2021; Nazy et al. 2021; Nguyen et al. 2017). Importantly, the presence of anti-PF4 auto-Ab has been associated with the development of thrombocytopenia and thrombosis following the ChAdOx1 nCoV-19 vaccine (AstraZeneca), presenting with clinical features commonly associated with HIT (Greinacher et al. 2021; Scully et al. 2021). The fact that only adenovirus-based COVID-19 vaccines are associated with HIT, suggest that other emerging viruses, may also trigger auto-Ab-mediated coagulopathy and auto-Ab screening is a potential tool in severe disease and vaccine risk surveillance.

### Autoantibodies and neurological disorders related to COVID-19

Emerging evidence suggest that a range of neurological outcomes are related to SARS-CoV-2 infection. While the cause of these neurological outcomes remains unclear, auto-Abs are increasingly being associated with COVID-19-related neuropathies. A recent study found diverse auto-Abs associated with Central Nervous System (CNS) diseases in eleven severe COVID-19 patients with neurological symptoms, such as aphasia, myoclonus, stroke, and epileptic seizure (Franke et al. 2021). In the patients’ serum, anti- N-methyl-D-aspartate (NMDA), cardiolipin, Beta-2 glycoprotein, Myelin, Yo, and Annexin auto-Abs were detected whereas only anti-Yo auto-Abs was observed in cerebrospinal fluid (CSF). Indirect immunofluorescence staining performed on unfixed murine brain incubated in CSF showed high intensity of IgG binding to vessel endothelium, perinuclear antigens, astrocytic proteins, neuropil of basal ganglia, and hippocampus or olfactory bulb. Although, the difficulty in obtaining CSF from individuals without neurological disorders limited the definitive conclusion of this study, CSF sample from all patients in the study were SARS-CoV-2 PCR negative, suggesting that neurological symptoms was not due to presence of the virus, but could be a result of autoimmune reactivity (Franke et al. 2021).

Similarly, evidence of association between Peripheral Nervous System (PNS) disorders and COVID-19 related auto-Abs has been observed. COVID-19 patients with bilateral paralysis and acute confusional state showed strong immunofluorescence staining in autoantibody screening using rat tissues (kidney, stomach and liver) incubated in serum from all patients (Schiaffino et al. 2020). In contrast, strong positive immunofluorescence signal was only detected when the tissues were incubated in CSF from Guillain Barre Syndrome (GBS) patient samples (Schiaffino et al. 2020). GBS is a condition in which the immune system attacks the peripheral nervous system causing demyelination and axon damage leading to muscle and body weakness. Previously, this syndrome has been associated with *Campylobacter jejuni*, Cytomegalovirus, Epstein-Barr virus (EBV) (Jacobs et al. 1998), and Zika virus (Cao-Lormeau et al. 2016; Leonhard et al. 2020) but since the start of the COVID-19 pandemic there have been 220 GBS-associated SARS-CoV-2 infection reported (Finsterer and Scorza 2021).

The underlying mechanisms for auto-Ab induction that potentially trigger neurology disorders related to COVID-19 remains elusive. However, molecular mimicry where pathogens have similar peptides or structures with self-antigens, triggering the immune system to attack self-antigens has been suggested to cause this occurrence (Al-Ramadan et al. 2021). Sequence analysis of SARS-CoV-2 and human proteins associated with neuropathies found two immunological peptides, KDKKKK and EIPKEE, identical with human heat shock protein 90-beta (HSP90B, HSP90B2) and 60 kDa heat shock protein mitochondria (HSP60), respectively (Lucchese and Floel 2020). However, a study conducted by Mantej et al*. *(2021) showed no significant difference in anti-HSP60, anti-HSP70, and anti-HSP90 auto-Ab levels in serum of SARS-CoV-2 seropositive patients, seropositive mRNA vaccinated individuals, and seronegative healthy individuals (Mantej et al. 2021). Collectively these findings suggest that auto-Abs are important in COVID-19-related neurological disorders. However, functional validation is essential to determine whether CSF auto-Abs are pathogenic or not.

### COVID-19 as a potential trigger for autoimmunity

Research suggests individuals with pre-existing co-morbidities, including auto-Abs, may be at greater risk of developing severe COVID-19. However, auto-Abs have been found in individuals without co-morbidities post-COVID-19 infection (Bastard et al. 2020; Wang et al. 2021; Jacobs 2021; Khamsi 2021). The assessment of whether SARS-CoV-2 infection triggers autoimmunity is hampered by the lack of availability of longitudinal samples pre- and post-COVID-19 from the same individuals. However, given that prevalence of auto-Abs is significantly higher in critically ill COVID-19 patients compared to healthy community controls, auto-Ab production may in some cases be induced de novo. In support of this, emerging reports using early time points after infection describe new onset auto-Abs in a subset of hospitalised patients (Chang et al. 2021; Reiff et al. 2021). As the significance of auto-Abs for severity of COVID-19 disease is emerging, auto-Abs have also been suggested to play a role in slow recovery during long COVID (Wallukat et al. 2021), but the full extent of their link to long COVID remains to be established (Khamsi 2021). Undoubtedly, more investigations into  both pre-existing and COVID-19- induced auto-Abs are needed to understand their relative contribution towards severe disease prognosis. Similarly, the role they play in long COVID will need to be thoroughly assessed to understand the factors leading to one of the major conundrums facing the global community.

## Conclusions

Measuring the presence of auto-Abs, opens up possibilities to screen for predispositions to severe COVID-19 prognosis in addition to demographical factors to identify patients at risk. This will provide fundamental information important for the advancement of treatment options and support individuals on a case-by-case basis during disease intervention and management. For example, therapeutics against hyperactivation of neutrophil-mediated tissue damage may be a candidate to mitigate severe COVID-19 disease (Nemeth et al. 2020). Currently, existing therapeutics that block auto-Ab receptors in neutrophils such as FcγR (Wang and Jonsson 2019) and complement receptor (Ricklin and Lambris 2007), may be useful to minimise lung tissue damage in ANCA-positive individuals by reducing NET production and disrupting the NETosis pathway. The presence of anti-PF4 associated with HIT suggests an alternate anticoagulant treatment to heparin should be used for COVID-19 patients or that patients are screened for auto-Ab prior to treatment. Similarly, auto-Ab screening of individuals may improve ChAdOx1 vaccine program safety or inform appropriate treatment in individuals who develop adverse thrombotic reactions. The emerging diverse role of auto-Ab in both the development of and protection from severe COVID-19, places auto-Ab at the forefront on risk surveillance in future viral epidemics.
